# Diagnostic copper imaging of Menkes disease by synchrotron radiation-generated X-ray fluorescence analysis

**DOI:** 10.1038/srep33247

**Published:** 2016-09-15

**Authors:** Miyuki Kinebuchi, Akihiro Matsuura, Tohru Kiyono, Yumiko Nomura, Sachiko Kimura

**Affiliations:** 1 Department of Molecular Pathology, Graduate School of Medicine, Fujita Health University, Toyoake, Aichi, 470-1192, Japan; 2 National Cancer Center Research Institute, 5-1-1Tsukiji, Chuo-ku, Tokyo, 104-0045, Japan; 3 Aomori City Public Health Center, 19-13 Tsukuda 2-chome, Aomori City, Aomori, 030-0962, Japan; 4 Hokkaido Medical Center for Child Health and Rehabilitation, 240-6 Kanayama 1-jo 1-chome, Teine-ku, Sapporo, Hokkaido, 006-0041, Japan

## Abstract

Copper (Cu) is an indispensable metal for normal development and function of humans, especially in central nervous system (CNS). However, its redox activity requires accurate Cu transport system. ATP7A, a main Cu^2+^ transporting-ATPase, is necessary to efflux Cu across the plasma membrane and synthesize cuproenzymes. Menkes disease (MD) is caused by mutations in *ATP7A* gene. Clinically, MD is Cu deficiency syndrome and is treated with Cu-histidine injections soon after definite diagnosis. But outcome of the most remains poor. To estimate the standard therapy, Cu distribution in the treated classic MD patients is analyzed by synchrotron-generated X-ray fluorescence technique (SR-XRF), which identifies and quantifies an individual atom up to at subcellular level of resolution with wide detection area. SR-XRF analysis newly reveals that Cu exists in spinal cord parenchyma and flows out via venous and lymph systems. By systemic analysis, excess Cu is detected in the proximal tubular cells of the kidney, the mucosal epithelial cells of the intestine, and the lymph and venous systems. The current study suggests that the standard therapy supply almost enough Cu for patient tissues. But given Cu passes through the tissues to venous and lymph systems, or accumulate in the cells responsible for Cu absorption.

Menkes disease (MD) is an X-linked recessive disorder, caused by mutation in the Cu-transporting ATPase gene (*ATP7A*)^1^,^2^. The ATP7A protein is a transmembrane P-type ATPase that binds Cu(I) delivered by Cu chaperons such as Atox 1, and translocates Cu within the *trans*-Golgi network (TGN) to incorporate it into cuproenzymes, or efflux Cu across the plasma membrane to maintain cellular Cu homeostasis^2^. Clinically, MD is Cu deficiency syndrome by featured the dysfunction of multiple Cu-dependent enzymes. One of the clear etiologies is malabsorption of Cu from intestinal tract^1^,^3^. At the onset of symptoms, the MD patients’ laboratory data show low levels of serum Cu and ceruloplasmin. Histidine-Cu injections immediately normalizes their levels^3^,^4^,^5^. Despite the treatment is started at pre-symptomatic stage, outcome of most of them remains still poor^6^,^7^. To clarify the results of the standard therapy, we analyzed the Cu status using core-shell electron-generated X-ray fluorescence (XRF), which is counted by the number of photons at atomic number specific X-ray frequency according to Moseley’s law^8^.

## Results

### A novel disease-causing mutation C1640T in the *ATP7A* gene in two unrelated Japanese patients with Menkes disease

**Figure 1:**
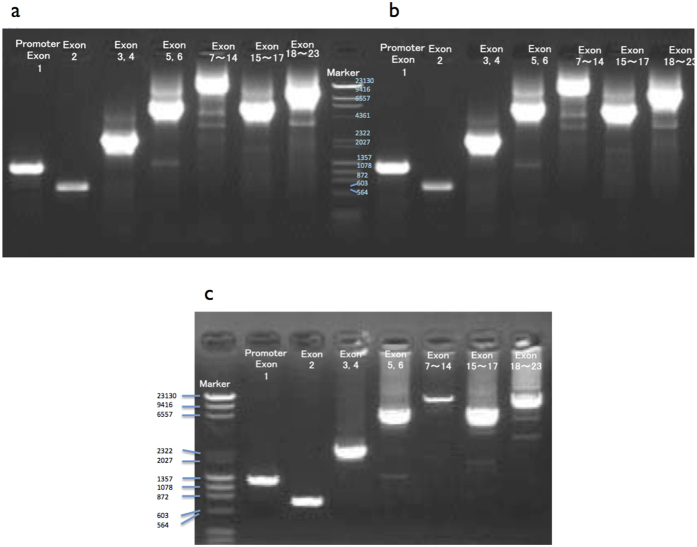
PCR amplification of ATP7A gene. Genomic DNA isolated from (**a**) an unaffected individual (control no. 1), (**b**) patient no. 1, and (**c**) patient no. 2. Template DNAs were extracted from cultured skin fibroblasts [(**a**) and (**b**)] and from splenic tissue (**c**). The amplified region of *ATP7A* gene is noted on the top of each lane. Sizes of the marker DNA fragments are shown in base pairs.

**Figure 2:**
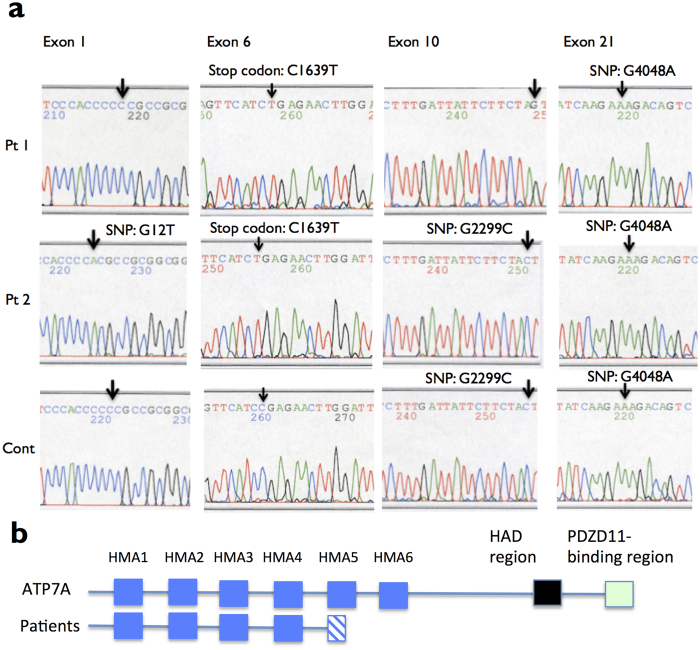
Mutational analysis of ATP7A gene. (**a**) *ATP7A* nucleotide sequences of PCR products from genomic DNA of patient no. 1 (Pt 1), patient no. 2 (Pt 2) and an unaffected individual (control no. 1) (Cont). NM_000052 (version 6) in GenBank provides the nucleotide number as reference sequence. In exon 1, patient no. 2 has a G12T substitution (arrow, complementary strand). In exon 6, patient no. 1 and patient no. 2 have a C1639T substitution, which resulted in stop codon as Arg547X (arrow). In exon 10, patient no. 2 and an unaffected individual have a G2299C substitution (arrow). In exon 21, all have a substitution G4249A (arrow). (**b**) Expected ATP7A protein. The ordinary ATP7A protein and the truncated mutant ATP7A protein in patients are aligned.

The direct sequencing of the PCR products of 23 exons, promoter region, and splicing donor and acceptor regions of the *ATP7A* gene in the 2 unrelated Japanese patients with classic MD and an unaffected control is done in this study. The each PCR product of them is similar size as the reference *ATP7A* gene (NM_000052) (Fig. 1). A total of 2 and 4 nucleotide substitutions are found in the patient no. 1 and 2, respectively (Fig. 2a). A nonsense mutation C1640T in exon 6 found in both patients results in premature stop codon Arg547X in the 5th copper-binding domain of ATP7A protein. This disease-causing mutation has not been identified previously^9^. The other 3 substitutions represent allelic variants, single nucleotide polymorphisms (SNPs); a substitution G to T in exon 1 at position 12 is found in patient no. 2, a missense mutation, G2299C in exon 10 (Leu767Val) is found in patient no. 2 and an unaffected control. Further, a missense mutation G4048A in exon 21 (Glu1350Lys) is found in two patients and an unaffected control by comparing with the reference sequences, NM_000052 and NM_001282224. A4048 encoding Lys1350 also exists in isoform 1 to 5 of Q04656 in UniProt. There is no nucleotide substitution in 5’UTR (about 600 bp), and splicing donor and acceptor of all exons.

### **MD derm**al keratinocyte accumulated Cu

**Figure 3:**
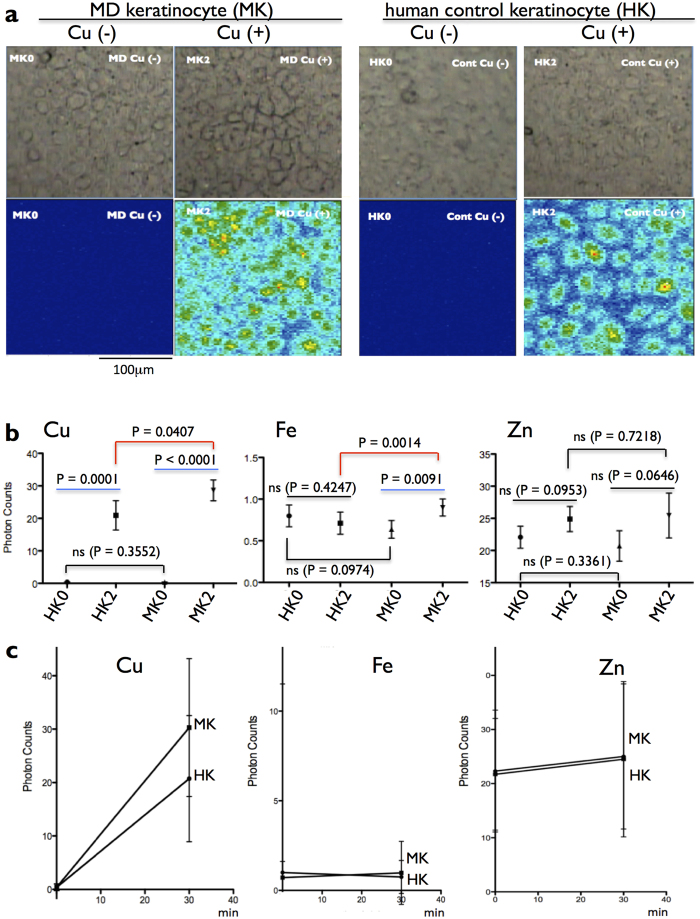
Effects of Cu loading on keratinocyte cell lines. (**a**) Effects of Cu loading on subcellular distribution of Cu in the cultured keratinocyte lines from an unaffected control (control no. 1) and the MD patient, HK and MK, respectively. (*Upper*) Analyzed area of culture inserts is shown using a stereoscopic microscopy. The culture density is adjusted. (*Lower*) 2-D imaging of Cu-Kα derived SR-XRF. Cu signal is not visible in Cu-untreated cells (HK0 and MK0). After Cu treatment (HK2 and MK2), Cu signals are detected in the cytoplasm and nuclei in MK2 more than HK2. A scale bar: 100 µm. (**b**) Effect of Cu loading on the kinetics of intracellular elemental contents (two-tailed paired t Tests). (**c**) Cu retention rate of MK is higher than HK. Cu-load oppositely effects on intracellular Fe kinetics of MK and HK. All data was calculated from three individual experiments (mean ± SEM, *P* < 0.05). Prism version 5.00 d software is used for the graphical statistical analysis.

The keratinocyte cell lines of the MD patient and an unaffected control are adjusted the culture density, and used at the day of the culture inserts being confluent (Fig. 3a). Before Cu treatment, the Cu content in MD keratinocytes is indistinguishable with control keratinocytes (P = 0.3552) (Fig. 3b). After treatment with 100 μM-CuCl_2_ for 30 min, Cu appears in the cytoplasm and nuclei in MD and control keratinocytes (DA = 200 μm^2^) (Fig. 3a), and Cu content in MD keratinocytes is significantly higher than control keratinocytes (P = 0.0407) (Fig. 3b). To compare cellular Cu kinetics, Cu retention rate is obtained by dividing the value obtained by subtracting the Cu-concentrations in untreated cells from those in treated cells by those in untreated cells. Cu retention rate of MD keratinocytes (73.67) is 1.4 times higher than control keratinocytes (53.57). Alternatively, the rate of change of the Cu content in the cells with or without Cu loading is graphical statistical analyzed (Fig. 3c). Each slope is perfect line of 95% confidence intervals, and each R square is 1.000. Best-fit values of 1/slope of control and MD keratinocytes are 1.474 and 1.003, respectively, which values are according to the calculated rate of change of the Cu content in the cells. When Cu is loaded, Fe content increases in MD keratinocytes (1/slope = 111.9, R square = 1.000), but decreases in control keratinocytes (1/slope = −119.1, R square = 1.000) (Fig. 3c). When Cu is loaded, Zn content increases slightly in control keratinocytes (1/slope = 11.13, R square = 1.000) and MD keratinocytes (1/slope = 10.76, R square = 1.000) (Fig. 3c).

### Concentrations of Cu, Fe and Zn detected by atomic absorption spectroscopy

**Figure 4:**
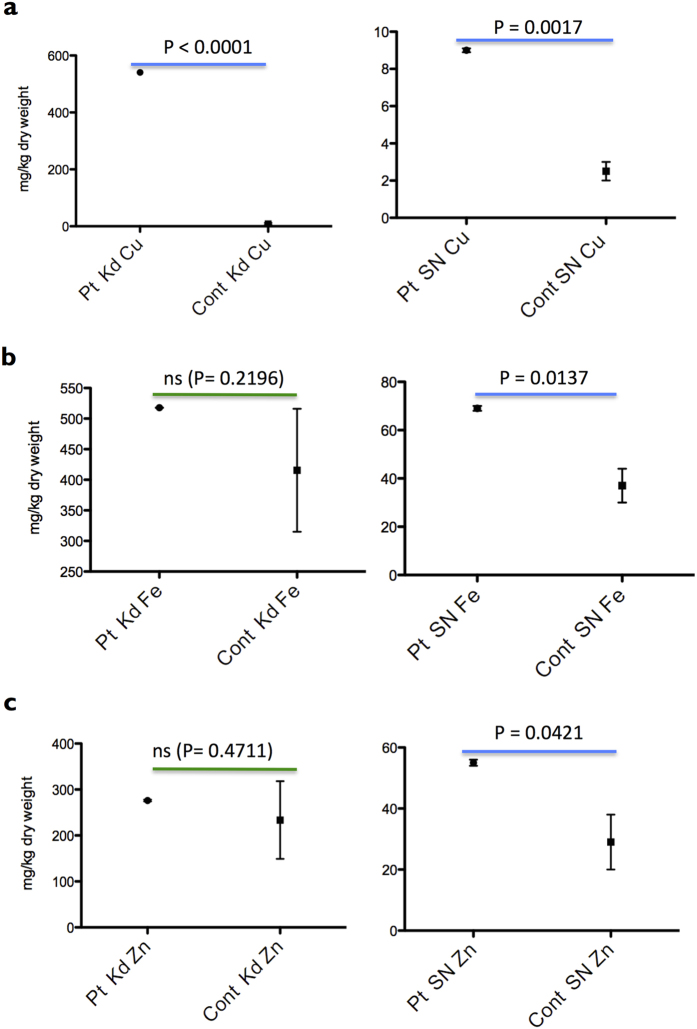
Cu, Fe and Zn concentrations in the kidney (Kd) and the spinal cord (SN) by atomic absorption spectroscopy. All data was calculated from three individual experiments (two-tailed paired t Tests). The kidney (Kd) and spinal cord (SN) derived from the MD patients (Pt) and the unaffected control (Cont) were subjected to the measurement of contents of copper (Cu), iron (Fe), and zinc (Zn) by atomic absorption spectroscopy (AAS, Shimadzu AA-6800). In this experiment, the spinal cord (SN) contains the root nerves, the vessels, and pia mater.

First we detect the elemental contents in organs by a Shimadzu atomic absorption spectroscopy AA-6800. In this experiment, the spinal cord (SN) contains the root nerves, the vessels, and pia mater. Cu concentrations in the patient’s kidney (Kd) and SN are significantly higher than the unaffected controls by two-tailed paired t Tests (P < 0.0001 and P = 0.0017, respectively) (Fig. 4a). Further, Fe and Zn concentrations in the patient’s SN are significantly higher than the unaffected controls (P = 0.0137 and P = 0.0421, respectively) (Fig. 4b,c). But Fe and Zn concentrations in the patient’s Kd are indistinguishable with the unaffected controls (P = 0.2196 and P = 0.4711, respectively) (Fig. 4b,c).

### Cu distribution in the gut

**Figure 5:**
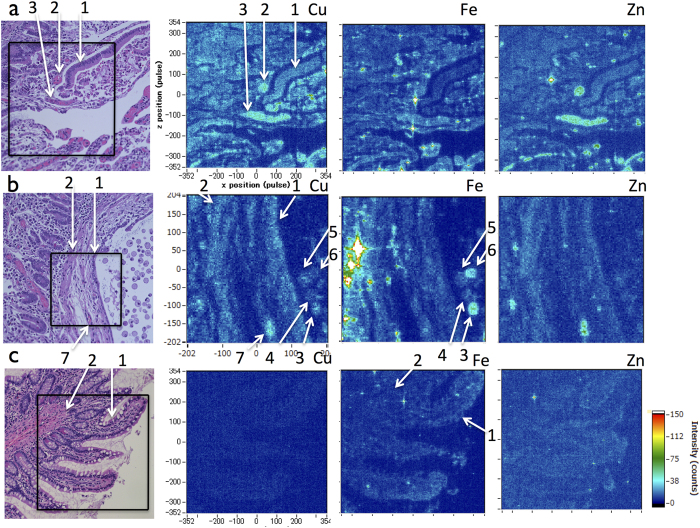
2D-imaging of subcellular Cu, Fe and Zn distribution in the intestinal tissues by SR-XRF analysis. (*Left*) The square provides the analyzed area of the serial section by SR-XRF (H & E staining). (**a**) The duodenum of the MD patient. High levels of Cu in the duodenal epithelium (arrow 1) and vessels (arrows 2 and 3) are obvious. (**b**) The lymph vessel enlargement in the duodenum of the patient. The arrows point the lymph endothelium (arrow 1), the inner muscular layer (arrow 2), macrophages (arrows 3 to 6) and vessel 3 (arrow 7). (**c**) Cu signals do not make any obvious image in the normal intestine derived from control no. 2. Arrow 1 and arrow 2 indicate epithelium and inner muscular layer, respectively. Similar results are shown in other controls. The element specific Kα are Cu (8 KeV), Fe (6.4 KeV), and Zn (8.6 KeV). Detected amounts of photon count (*y*) at a location (*x, z*) are expressed according to a scale of the rainbow-color of the right bar. (Scale bar, 100 μm). Representative areas and similar areas were scanned at least three times.

**Figure 6:**
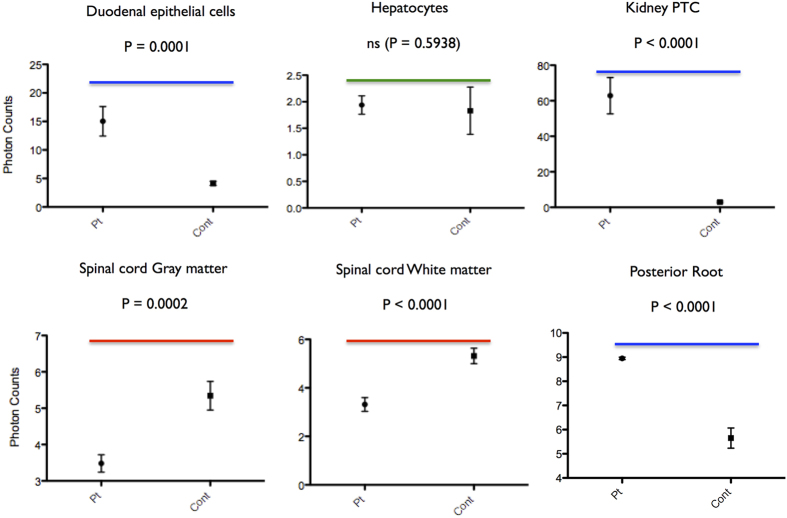
Cu concentrations (photon counts/0.5 sec) in tissues detected by SR-XRF between Cu-treated MD patients versus unaffected individuals. P = two-tailed paired t Tests. Six regions inside each tissue are calculated. Exceptionally, whole posterior root is calculated. All data are analyzed from three individual SR-XRF experiments.

The biopsied specimen of duodenum of MD patient no. 1 is stained with hematoxylin-eosin, and the squares provide the analyzed area of the serial section by SR-XRF. Intracellular Cu content of patient’ duodenal epithelium is high (Fig. 5a, arrow 1; 13.21 ± 3.85 photons/0.5 sec, Table 1). Further, Cu content of the vessels of duodenum reveals almost 2-fold high as compared with duodenal epithelium (Fig. 5a, arrows 2 and 3; 26.47 ± 6.68 and 26.12 ± 6.49 photons/0.5 sec, respectively, Table 1). Zn content of the vessels is obviously high as well (Fig. 5a, arrows 2 and 3; 50.95 ± 8.72 and 45.47 ± 8.62 photons/0.5 sec, respectively, Table 1). Next, the region including enlarged lymph vessel in the section of the duodenum is focused (Fig. 5b). It shows moderate high Cu content of the lymph endothelium (Fig. 5b, arrow 1) and the inner muscular layer (Fig. 5b, arrow 2) (11.05 ± 4.15 and 11.15 ± 3.87 photons/0.5 sec, respectively, Table 1). In more detail, the contents of elements of the macrophages in lymph vessels of duodenum are different to each other (Fig. 5b, arrows 3, 4, 5 and 6, Table 1). In contrast, biopsied specimens of the intestine of the unaffected controls show any significant image by Cu (Fig. 5c). Cu content by photon counts of the patient’s duodenal epithelium is significantly higher than the unaffected controls (P = 0.0001, Fig. 6).

### Cu distribution in the kidney

**Figure 7:**
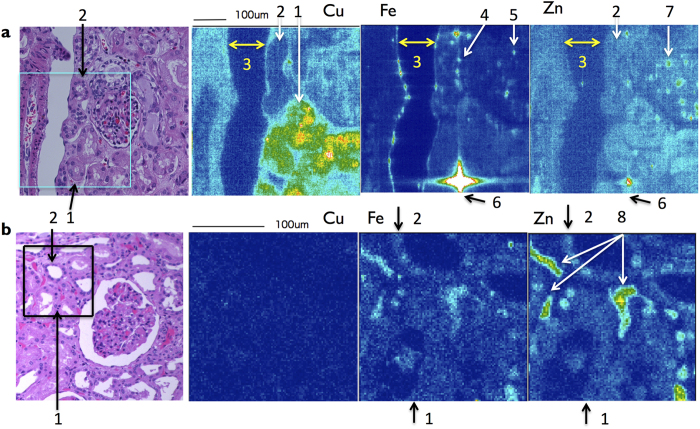
2D-imaging of subcellular Cu, Fe and Zn distribution in the kidney by SR-XRF analysis. (**a**) The cortex area of the kidney in the patient. A high level of Cu in PTC (arrow 1) and a low level of Cu in DTC (arrow 2) are shown. The interlobular arterial wall (arrow 3), Bowman’s capsule (arrow 4), mesangium (arrow 5) and a stone (arrow 6) are visible by Fe signals. A high level of Zn in the glomerulus capillary is corresponding to red blood cells (arrow 7). (**b**) The kidney cortex of an unaffected control no. 3. PTC and DTC are arrow 1 and arrow 2, respectively. Arrows 8 indicate vessels. Cu does not make any obvious image, and similar results are shown in other controls. High levels of Fe and Zn signals are visible in vessels. The square provides the analyzed area of the serial section by SR-XRF (H&E staining). Representative areas and similar areas were scanned at least three times.

Figure 7a shows SR-XRF analysis of kidney tissue of the patient. The proximal tubular cells (PTC) are shown by quite high photon counts of Cu (Fig. 7a, arrow 1; 56.71 ± 11.72 photons/0.5 sec, Table 2), where an excess Cu is detected in the nucleus and the cytoplasm. In contrast, the level of Cu of the distal tubular cells (DTC) of the patient is not high (Fig. 7a, arrow 2; 9.75 ± 3.18 photons/0.5 sec, Table 2), similar as the unaffected control (Fig. 7b, arrow 2; 9.85 ± 1.31 photons/0.5 sec, Table 2). The interlobular arterial wall of the patient is shown by Cu signals (Fig. 7a, arrow 3; 17.31 ± 4.15 photons/0.5 sec, Table 2). Fe is condensed in bowman’s capsule (Fig. 7a, arrow 4; 61.2 ± 7.30 photons/0.5 sec, Table 2), mesangium (Fig. 7a, arrow 5; 55.78 ± 31.56 photons/0.5 sec, Table 2), and interlobular arterial wall (Fig. 7a, arrow 3; 36.30 ± 28.21 photons/0.5 sec, Table 2) in the patient. Zn content in PTC (25.42 ± 4.89 photons/0.5 sec, Table 2) is higher than DTC (18.88 ± 4.39 photons/0.5 sec, Table 2) in the patient. High Zn spots (55.54 ± 7.44 photons/0.5 sec, Table 2) in the glomerulus are corresponding to red blood cells (Fig. 7a, arrow 7) in the patient. In contrast, Fig. 7b shows no significant accumulation of Cu and Fe in PTC and DTC in the kidney of unaffected control no. 3 (Fig. 7b, Table 2). Similar results are shown in other controls. Cu content of the patient’s PTC is significantly higher than the unaffected controls (P < 0.0001, Fig. 6).

### **Cu d**istribution in the spinal cord

**Figure 8:**
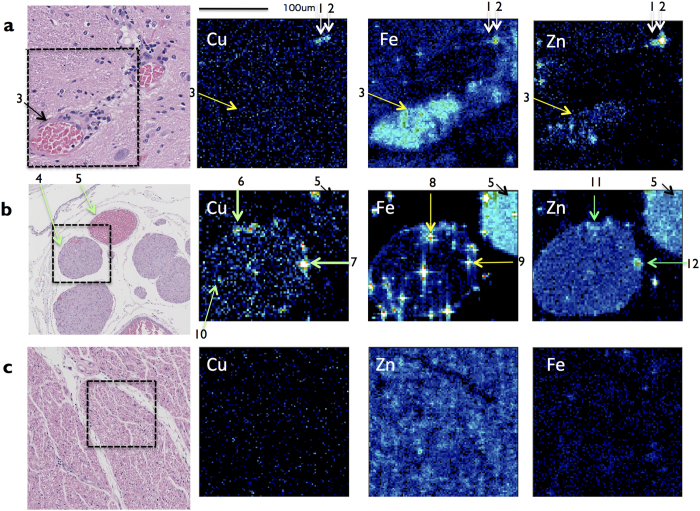
2D-imaging of subcellular Cu, Fe and Zn distribution in the spinal cord by SR-XRF analysis. (*Left*) The square provides the analyzed area of the serial section by SR-XRF (H & E staining). (**a**) Gray matter of the spinal cord in the patient. A high level of Fe pointed by arrow 3 is the branch of artery of central sulcus. White arrows indicate region 1 and 2. (**b**) Arrow 4 and arrow 5 indicate the posterior root and posterior spinal vein, respectively. Arrows 6 and 7 indicate Cu-high regions. Arrows 8 and 9 indicate Fe-high regions. Arrows 11 and arrow 12 indicate Zn-high regions. Arrow 10 indicates one of dot-like Cu-high regions. (**c**) Cu signals do not make any obvious image in the normal posterior root. Nerve region is oriented by Zn signals. The element specific Kα are Cu (8 KeV), Fe (6.4 KeV), and Zn (8.6 KeV). Detected amounts of photon count (*y*) at a location (*x, z*) are expressed above average photon counts according to a scale of the rainbow-color. (Scale bar, 100  µm). Representative areas and similar areas were scanned at least three times.

SR-XRF analysis reveals that gray matter (GM) and white matter (WM) of spinal cord of the patients contain Cu (3.48 ± 0.24 photons/0.5 sec and 3.32 ± 0.28 photons/0.5 sec, respectively, Table 3), although the levels of Cu of them are significantly lower than the unaffected controls (P = 0.0002 and P < 0.0001, respectively, Fig. 6). Figure 8a shows SR-XRF analysis of spinal cord tissue of the patient. A high level of Fe (62.91 ± 7.61 photons/0.5 sec), and a moderate level of Zn (23.91 ± 4.66 photons/0.5 sec) in the branch of artery of central sulcus are shown (Fig. 8a, arrow 3, Table 3). However, the level of Cu in the artery is not higher than the gray matter of the patient (Fig. 8a, arrow 3 ± 1.98 photons/0.5 sec, Table 3). Further, we couldn’t detect the Cu accumulation in blood endothelium within the analyzed area of spinal cord of the patient. In the gray matter of the patient, two regions contain high amount of Cu (Fig. 8a, arrows 1 and 2, 17.25 ± 2.99 and 16.50 ± 5.45 photons/0.5 sec, Table 3).

Cu content of whole posterior root of the patient is significantly higher than the unaffected controls (Fig. 8b, arrow 4; 8.95 ± 0.05 photons/0.5 sec, Table 3; P < 0.0001, Fig. 6). Two regions inside the posterior root of the patient contain extremely high amount of Cu (Fig. 8b, arrows 6 and 7). The high level of Fe- and the high level of Zn-regions are shown near the two Cu^high^ regions, (Fig. 8b, arrows 8, 9, 11 and 12). Cu content of root nerve region (RN) is calculated independently to exclude the two Cu^high^-regions. The root nerve region of the patient contains Cu more than the unaffected controls (P = 0.0014). Inside the patient’s root nerve region, many small regions contain Cu more than the patient’s posterior spinal vein (Fig. 8b, arrows 10 and 5, P = 0.0070).

### Analysis of vessels by elemental composition

**Figure 9:**
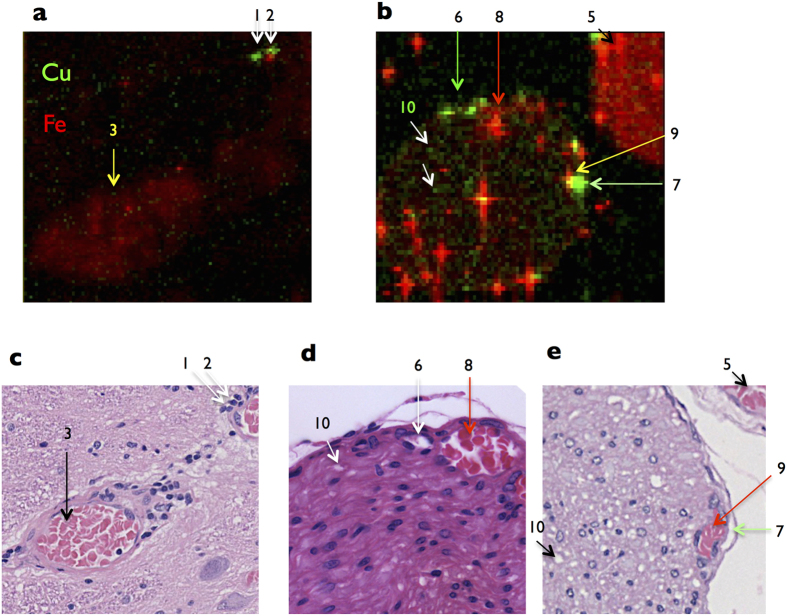
Distinct localization of Cu and Fe. 2D-images by SR-XRF analysis are merged in a coordinate. Cu- and Fe-signals are shown by green and red density. (**a**) Gray matter in the section. Arrows 1 and 2 indicate Cu^high^ regions. Arrow 3 indicates Fe^high^ vessel. (**b**) Arrow 5 indicates the posterior vein. Arrows 6 and 7 indicate Cu^high^Fe^low^ regions. Arrows 8 and 9 indicate Fe^high^ regions. Arrow 10 indicates one of dot-like Cu^high^ regions. (**C**) Arrows 1 and 2 indicate the gaps besides the small vessel. Arrow 3 indicates the branch of artery of central sulcus. (**d**) Arrow 6 and arrow 8 indicate the gaps besides the small vein and the small vein, respectively. (**e**) Arrow 7 and arrow 9 indicate the gaps besides the small vein and the small vein, respectively. Arrow 10 indicates the gap between nerve fibers. Each arrow indicates same region in Figs 8 and 9. Cu- and Fe-derived fluorescence X-ray is detected at Kα 8KeV and Kα 6.4 KeV, respectively. Representative areas and similar areas were scanned at least three times.

The levels of photon counts of Cu and Fe are shown in green and red density, and merged in a coordinate (Fig. 9). The region numbers in Figs 8 and 9 denote to same regions. The branch of artery of central sulcus is Fe^high^Cu^low^ (Fig. 9a, arrow 3). In the gray matter, a Cu^high^ small region is Fe^low^ (Fig. 9a, arrow 1). The other Cu^high^ small region exists besides Fe^high^ region (Fig. 9a, arrow 2). The Cu^high^ small regions seem to be corresponding with the gaps in gray matter (Fig. 9a, arrows 1 and 2, Fig. 9c, arrows 1 and 2).

In the posterior root, two Fe^high^ regions are veins (Fig. 9b, arrows 8 and 9). The highest level of Cu in Fe^high^ region indicated by arrow 9 is shown (Fig. 9b, Table 3). Since Cu^high^ regions indicated by arrows 6 and 7 are not Fe^high^ (Fig. 9b, Table 3), these regions exist besides veins (Fig. 9d, arrow 6 and Fig. 9e, arrow 7). Inside the patient’s root nerve region, a high level of Cu in many quite small regions mainly do not co-localize with Fe^high^ regions (Fig. 9b, arrows 10, Table 3). These Cu^high^Fe^low^ regions may be corresponding to the gaps between root nerve fivers.

## Discussion

Copper (Cu) is an essential trace element for every human cell, mainly for multiple Cu-dependent enzymes^1^,^3^. Because Cu ion is toxic with the redox activity simultaneously, it exists in organisms as a complex with a series of chaperons and transporters^1^,^2^. However, Cu itself in human tissues has not been observed clearly at elemental level. To clarify Cu status, the pathological specimens are irradiated by monochromatized microbeam X-ray at beamline BL37XU of SPring-8 and Photon Factory of KEK, which brilliance is up over 2.3 × 10^20^ (photons/sec/mm^2^/mrad^2^/0.1% bandwidth) by the inserted in-vacuum type undulator. The emitted core-shell electron generated X-ray fluorescence (XRF) is counted by the number of photons at atomic number specific X-ray frequency.

Menkes disease (MD) is caused by mutation in the *ATP7A* gene. Here we show two unrelated Japanese male patients characterized by typical phenotype of classic MD. Genomic DNA analysis reveals that both have C1640T in exon 6 of the *ATP7A* gene, which converts Arg547 to a stop codon within the 5th Cu-binding domain in the N-terminal (Fig. 2). Because the *ATP7A* gene locates on Xq21.1, both male patients do not have any functional ATP7A protein. ATP7A is one of two Cu^2+^-transporting ATPases of human, and expressed in most kinds of cells. ATP7A with eight transmembrane domains usually exists in the membrane of TGN, and delivered Cu^2+^ by ATOX1. Then, ATP7A handles Cu for biosynthesis of several Cu incorporated-proteins. When intracellular Cu content increases, ATP7A locates to plasma membrane to export Cu via the membrane to bloodstream^1^,^2^. We check the cellular Cu export ability of dermal keratinocyte cultures derived from our MD patient and the unaffected control same as mutational analysis. After treatment with 100 μM-CuCl_2_ for 30 min, Cu signals are detected in the cytoplasm and nuclei of MD and control keratinocytes by SR-XRF analysis (Fig. 3a). Retention rate of intracellular Cu of MD keratinocytes is 1.4 times higher than control keratinocytes (Fig. 3c). Our data indicate the dysfunction of Cu export ability of ATP7A in our MD patients. Unlike the several past experiments of fibroblasts^10^,^11^, Cu content is not differentiated without Cu treatment in MD keratinocytes and control keratinocytes (P = 0.3552) (Fig. 3b). The fundamental data reported by Goka that cultured MD skin fibroblasts had a concentration of Cu 5 times more than that of normal fibroblasts without Cu treatment^11^. They detected Cu and protein concentrations by atomic absorption spectrophotometry and UV absorption method, respectively. Then, the Cu concentrations were divided by the protein concentrations of the cultures, since the protein concentrations were proportional to culture size in their system. Thus, the protein concentrations per cell might affect on the calculation results in their equation. Alternatively, the Cu concentrations of MD keratinocytes might be different from fibroblasts.

After treatment with CuCl_2_, Fe significantly increases in MD keratinocytes (Fig. 3b). This is according to ^59^Fe accumulation in *Atp7a* knockdown rat intestinal epithelial (IEC-6) cells in culture^12^. ATP7A may involve in cellular Fe-homeostasis.

Next the elemental concentrations in organs are detected by AAS (Shimadzu AA-6800). Total amount of Cu in the patient’s Kd (kidney) and SN (spinal cord, root nerves, the vessels, and pia mater) are significantly higher than the unaffected controls (Fig. 4a). In addition, Fe and Zn contents in the patient’s SN are significantly higher than the unaffected controls (Fig. 4b,c). Because normal level of serum ceruloplasmin of the patients was maintained under Cu-histidine treatment, we speculate that a high level of Fe may be caused mainly by several times of blood transfusions for the patients. Then, we contribute SR-XRF analysis to make it clear where the excess Cu exists.

In humans, the initial Cu acquisition is the absorption of dietary Cu (reference range 0.6–2.0 mg/day) across the intestinal epithelium, where ATP7A flow out Cu into the blood vessel from the basolateral membrane of mucosal cells^1^,^2^. A series of our SR-XRF analyses always show any significant image of the gastrointestinal tissue derived from unaffected individuals by Cu levels (Fig. 5c). SR-XRF analysis reveals the Cu accumulation in the mucosal epithelial cells and the vessels in the duodenum of the MD patient (Fig. 5a). The level of Cu in the vessels is twice high as compared with the epithelial cells (Table 1). Our data supports previous studies by atomic absorption spectroscopy of a high level of Cu in the intestinal tissues of MD patient^13^ and Cu-treated animal models^14^. Since the level of Cu is also high in the lymph endothelium, the inner muscular layer and some macrophages in the lymph of the duodenum of the patient (Fig. 5b), we suppose that Cu is released from the degenerated epithelial cells into interstitial fluid, then to flow into the vessels and the lymph in duodenum. Dietary Cu enters into intestinal epithelial cells via apical membrane. Injected Cu in vessels enters into intestinal epithelial cells via basal membrane by Ctr1. The variety of elemental contents (Cu, Zn, Fe) of these macrophages is supposed to be the result of phagocytosis. In the patient, diffuse hemorrhagic erosion in the duodenum has been shown by duodenoscopy. Thus, excess Cu is released into the digestive tract as fallen off mucosal cells as well. SR-XRF reveals a high level of Zn in the same Cu^high^-vessels, which seems to be corresponding with red blood cells (Fig. 5a).

In healthy human, dietary absorbed Cu is circulated as albumin- and transcuprein-binding forms mainly to the liver through portal vein and partially to the kidney^1^,^15^. Unlike this in the patient, injected Cu-histidine enters the bloodstream from the capillaries and lymphatic. This treatment normalized serum Cu and celuroplasmin levels of our patients. Supportively, Cu-histidine is up-taken by hepatocytes efficiently^5^. SR-XRF analysis reveals that Cu content in the patient’ hepatocytes is indistinguishable with controls (P = 0.5938) (Fig. 6). The results suggest that ATP7A is not a main Cu transporter in the hepatocytes. Another P-type ATPase, ATP7B incorporates Cu into ceruloplasmin, known as ferroxidase and a major carrier of Cu to the body via circulation in the hepatocytes. Further, ATP7B exports excess Cu to bile^2^.

In healthy human, Cu is excreted mainly via bile and small amount of it via urine. SR-XRF analysis of the patients’ kidney shows a quite high level of Cu in the proximal tubular cells (PTC) (Fig. 7 arrow 1, Fig. 6, and Table 2). A high level of Cu in PTC of the patient may be caused by defective Cu re-absorption mechanism in the kidney, and enhanced by Cu-histidine treatment. Urinary Cu is absorbed in PTC, and excreted from the basolateral membrane into blood circulation by ATP7A^16^. Without ATP7A dysfunction, acute Cu-toxicosis affects on PTC, and results in acute tubular necrosis^17^,^18^. Chronic Cu toxicosis caused by ATP7B deficiency named Wilson’s disease, affects on primary the liver and at last PTC^5^,^16^. Taken together, the interstitial nephritis of this patient is explained as Cu toxicosis on PTC. In addition, high levels of Fe and Cu in Bowman’s capsule and mesangium cells may be the results of phagocytosis (Fig. 7, Table 2).

CNS including spinal cord has separated circulation system from body by blood-brain barrier (BBB) and blood-cerebrospinal fluid barrier (BCSFB). Tight junctions of lining vascular endothelial cells and associated astrocytes of CNS form the BBB, where ATP7A existence on basolateral membrane of vascular endothelial cells is essential for Cu transport into interstitial fluid (ISF)^19^. Cu is an indispensable metal for normal development and function of CNS^6^. Thus, most serious symptom of MD is caused by Cu deficiency of CNS^1^,^5^. The original our question is whether Cu exists in patient’s spinal cord parenchyma. SR-XRF analysis reveals that gray matter and white matter of spinal cord contain Cu, although the amounts are lower than the unaffected controls (Fig. 6). Cu enters into CNS possibly via BCSFB, where the BBB is not formed. At the choroid plexus (CP), the BCSFB is formed by tight junctions of lining CP epithelial cells, and Ctr1 on blood-side membrane of CP and ATP7B on CSF-side membrane of CP transport Cu from blood to cerebrospinal fluid (CSF) circulation^19^. CSF flows through the ventricular cavities and subarachnoid space of brain and spinal cord. CSF is exchanged rapidly with ISF in animal brain^20^. The flow has a role of nutrient expansion. Thus, Cu could be expanded through a flow of CSF. Supportively, a recent alternative trial of *ATP7A* gene addition to CP results in better outcome in a MD mouse model^21^.

Because Cu amount in the patient’s spinal cord is higher than the unaffected controls by means of AAS (P = 0.0017) (Fig. 4), it is supposed that Cu may accumulate in the artery. However, SR-XRF analysis reveals a low level of Cu in the artery in gray matter. Furthermore, Cu is not accumulated in the blood endothelium in gray matter (Fig. 8a). A hypothesis is that a quite high level of Fe in the artery might associate with Cu transport mechanism of the blood endothelium in the patient’ spinal cord. By superimposing the levels of Cu and Fe in a two-dimensional image, the levels of photon counts of Cu and Fe are compared in a coordinate (Fig. 9). The branch of artery of central sulcus is Fe^high^ Cu^low^ (Fig. 9a, arrow 3). Newly, it reveals Cu^high^ Fe^low^ region in the gray matter (Fig. 9a, arrow 1), which seems to be corresponding with the gap in gray matter (Fig. 9c, arrow 1). The other Cu^high^Fe^low^ small region exists besides Cu^high^Fe^high^ region (Fig. 9a, arrow 2).

A significant high level of Cu in the patient’s posterior root is revealed by SR-XRF analysis (Fig. 8b, arrow 4). The regions indicated by arrows 8 and 9 show a high level of Fe, which are veins (Figs 8b and 9b). A vein indicated by arrow 9 show an extremely high level of Cu (Fig. 9b,e; Table 3). The other vein indicated by arrow 8 show a high level of Cu (Fig. 9b,e; Table 3). Further, the Cu^high^ regions exist besides veins (Fig. 9b, arrow 6 and arrow 7). The regions indicated by arrows 6 and 8 are separated by elemental photon counts. However, the regions indicated by arrows 7 and 9 are not separated clearly.

By more detailed analyses, inside the patient’s root nerve region, many small Cu^high^Fe^low^ regions exist (Fig. 9b, white arrows 10). Root nerve region consists of root nerve fibers and gaps between root nerve fibers. Thus, these small Cu^high^Fe^low^ regions may be gaps between root nerve fibers (Fig. 9d,e, arrow 10), which contain Cu more than the posterior spinal vein of the patient (Table 3).

Since the levels of Cu in the veins and gaps inside posterior root are extremely high, it is newly suggested that significant amount of Cu flows out via the posterior root from spinal cord. Previously the common clearance vessels of CNS are considered to be the veins in brain parenchyma and in dura mater^22^. Here we postulate that gaps besides vessels and between nerve fibers inside posterior root may work as lymph system.

In conclusion, we develop a new method to show two-dimensional distribution of elements in a pathological section using SR-XRF analysis. The advantage of it is the absolute specificity for the element and subcellular level of resolution with a significant detection area.

## Methods

### Participants

All participants provided an informed consent and the study protocols were approved by the Ethical Committee of Fujita Health University (No. 06–051, No. 09–064 and No. 15–147). The methods were conducted in accordance with approved guidelines; patients’ clinical data and all samples were obtained anonymously from medical records and given the original numbers on the date of the study. Preparation of Cu-histidine complex was according to the standard protocol in Japan^4^. Tissue samples were flash-frozen using liquid nitrogen within 2 h of biopsy and autopsy at −80 degree until analyses.

#### Patients

Two unrelated Japanese males were diagnosed as classic Menkes disease (MD) by clinical features, biochemical test results and clinical imaging, then received Cu-histidine treatment at 6-month-old and 4-month-old, respectively.

#### Control subjects

Four unaffected boys (n = 4) with age matched (neonatal to 4-year-old) were studied. Control No. 1 was subjected for immortalization of dermal keratinocytes and genomic DNA analysis. Controls No. 2, No. 3 and No. 4 were subjected for AAS and SR-XRF analyses.

### Case reports

#### Patient No. 1

He was born at 38 weeks by normal vasinal delivery weighing 3314 g. Funnel chest, sursumergence and flat back of the head were found since birth. Although brain computed tomography (CT) at 3-month-old showed no abnormality, obvious hypotonia progressed since 4-month-old. On first admission at 5-month-old he was somnolent with kinky hair, difficulty in breathing, and failure to thrive. High level of urinary Cu (168 μg/dL: reference range, 14–63 μg/dL), low levels of serum ceruloplasmin (8.6 mg/dL: reference range, 21–37 mg/dL) and serum Cu (39 μg/dL: reference range, 78–131 μg/dl), were pointed out. He had a normal karyotype. Electroencephalography (EEG) with focal seizure onset revealed focal epileptiform discharges in the posterior region. Brain magnetic resonance imaging (MRI) at 6- and 7-month-old revealed progressive atrophy of bilateral temporal lobes and a meandering of circle of Willis. At 6-month-old, a diagnosis of classis MD was made and injection of Cu-histidine (750 μg/day = 107 μg/kg/day, everyday) was started. Within a week, his serum levels of Cu and ceruloplasmin recovered to reference range. Then, Cu-histidine was reduced (same calculated-dose, twice a week). At 7-month-old, he developed west syndrome and was received sodium valproate (150 mg/day) with significant improvement. Then he was an outpatient with injection of Cu-histidine (same dose) and several times blood transfusion for anemia. He manifested progressive growth and psychomotor delay with sparse hypo-pigmented hair. At 4.8-year-old, he developed liver dysfunction and renal failure. At 4.9-year-old, he died of diffuse alveolar damage caused by massive hemorrhage from upper gastrointestinal tract.

#### Patient No. 2

He was born at 34 weeks gestation by cesarean operation weighing 2188 g. Apgar score was 7 and 8 after 1 and 5 minutes, respectively. At 35 days of age, his body weight was 2500 g. At 4 months old, he developed interstitial pneumonia and pneumothorax showed by chest radiographs, and developed focal clonic seizures, and then subsequently hospitalized. On admission, the low levels of serum ceruloplasmin (2.9 mg/dL), serum Cu (9 μg/dL), and urinary Cu (6.8 μg/day) were pointed out. He had a normal karyotype. Brain MRI showed atrophy with diffuse delayed myelination and a meandering of circle of Willis. With these findings, a diagnosis of classic MD was made at 6-month-old. Subsequently injection of Cu-histidine (90 μg/kg/week) was started. His serum levels of Cu and ceruloplasmin recovered to reference range. Brain MRI at 13-month-old showed progressive atrophy and meandering of vessels. Then, Cu-histidine was increased (900 μg/day, twice a week). A progressive esophageal hiatal hernia was treated surgically at 17-month-old. He developed acute hemorrhagic pneumonia and was received anti-fungal agents, antibacterial drugs, steroid and blood transfusion. At 18-month-old died of dyspnea.

### Synchrotron-generated X-ray fluorescence technique (SR-XRF)

The x-ray fluorescence of the specified energy, which corresponds to the energy of core-shell electron bonding of an element^9^,^23^, is counted by photons. Synchrotron radiation analyses were performed at the BL37XU beamline in the SPring-8 (JASRI) and Photon Factory (KEK)^24^,^25^,^26^,^27^. A monochromatized X-ray is focused to a spot size of 1.3-μm (vertical) x 1.3-μm (horizontal) with a measured flux of more than 10^11^ photons/sec at 10 KeV using Pt (ρ = 21.4 g/cm^3^)-coated K-B mirror optics at a glancing angle of 2.8 mrad. The formalin-fixed paraffin-embedded (FFPE) tissue with 2-μm thickness was mounted on the XY-scanning stage at a takeoff angle of 10 degree. The incident intensity of the beam (*I*_*0*_) was recorded for normalization. The X-ray fluorescence was detected with a single-element silicon drift detector (SSD; Roentec, Berlin, Germany) along with energy dispersive spectrometry (XEDS) by setting regions about the respective X-ray emission lines (Fe Kα; 6.4 KeV, Cu Kα; 8.0 KeV, and Zn Kα; 8.6 KeV). The scanning ranges were tuned at 1000 μm squares with 5  µm steps, 500  µm^2^ squares with 5 μm steps, and 200 to 100  µm^2^ with 1.5 to 1 μm steps. Thus, the pixel size (PS) with 10 μm × 10 μm and detection area (DA) with 1000  µm^2^ was used to detect fluorescence X-ray signals. To distinguish the observation objects at the subcellular level, the PS 2.5 μm × 2.5 μm and DA 220  µm^2^ was used. The data were collected for 0.5 sec/pixel. The position of the pathological specimen was expressed in two-dimensional coordinates (*x, z*). In a particular fluorescence X-ray energy specific for an element, the detected amounts of photon counts (*y*) were expressed according to a scale of the rainbow-color bar at a two dimensional point (*x, z*). The resultant visible three-dimensional model of the sample was made using LabView (National Instruments Japan, Tokyo) and IGOR pro (WaveMetrics, Inc. Lake Oswego USA).

#### Samples for SR-XRF

Biopsied and autopsied tissues derived from the patients and controls no. 2, no. 3 and no. 4 were analyzed by SR-XRF.

### Genomic DNA Analysis

Genomic DNA was extracted from cultured skin fibroblasts of patient no. 1 and control no. 1, and the splenic tissue of patient no. 2 as described^28^. Polymerase chain reaction (PCR) amplification using LA Taq DNA Polymerase (Takara, Japan) with a GeneAmp PCR System 2700 (Applied Biosystems, Foster, CA) were performed in the 5′-upstream region, each of the 23 exons, and the adjacent intronic sequences of the *ATP7A* genes. All of the PCR products were analyzed with electrophoresis on agarose gels, and directly sequenced with a 3700 DNA analyzer (PE Applied Biosystems, Foster City, CA) using a DNA sequencing kit (BigDye^TM^ Terminator Cycle Sequencing v2.0 Ready Reaction, ABI PRISM, Life Technologies) according to standard protocols.

#### Samples for genomic DNA analysis

Genomic DNA of the patient no. 1, patient no. 2 and control no. 1 were analyzed.

#### Immortalization of dermal keratinocytes

Dermal keratinocytes obtained from skin biopsies of MD patient no. 1 at 6-month-old and control no. 1 were introduced hTERT and human papilloma virus E6E7 to immortalize, named MK (MNK-HDF-TERT-E6E7 in the experimental record) and HK (HDK1-TERT-E6E7 in the experimental record), respectively. Both were established as described^29^.

### Copper accumulation test with dermal keratinocytes

Cells were grown using a defined keratinocyte-SFM kit (Gibco, Life Technologies) with penicillin, and streptomycin in 12 well cell culture inserts (0.4 micron high pore density PET, #3495, BD Falcon Japan, Tokyo) with 12 well plates (#3503, BD Falcon Japan) in a 5% CO_2_ at 37 degree Celsius incubator. When the cells became confluent, each well was rinsed twice with 1 ml phosphate buffered saline without Mg^2+^, Ca^2+^ (PBS (−)), then incubated in 1 ml Hanks buffered solution modified (HBSS, Sigma H6648) for 30 min in 5% CO_2_ at 37 degree. Then the cultures were added CuCl_2_ (Sigma C3279) in HBSS consisting of copper 100 μM (HK2 and MK2) or without copper (HK0 and MK0) and incubated for 30 min in 5% CO_2_ at 37 degree. Each well was rinsed twice with 1 ml PBS (−), and then added 1 ml of 4% paraformaldehyde solution for 5 min at room temperature. Following three washes with double-distilled water to remove extracellular elements in the buffer, the cells on the culture inserts were air-dried. Each analyzed culture insert is shown using a stereoscopic microscopy (SZX10, Olympus, Japan). The copper contents of cultured keratinocytes are represented as the mean of the average photon counts of the detection area (DA) with 1000  µm^2^ per 0.5 sec at 10 KeV. Copper contents of them were directly determined by SR-XRF analysis using BL37XU beamline. Statistical analysis for rate of change in the intracellular element (Cu, Fe, and Zn) is performed with GraphPad Prism 5.0 (GraphPad Software, Sandiego, CA).

#### Cu concentration and cell viability

For *in vitro* experiments, it has been generally accepted that the toxicity of copper in the culture medium exerted approximately milimolar level^14^, although the toxicity is actually depend on the cell types and primary or established cell lines. Previous reports by Petris used about 200 micromolar concentration of CuCl_2_ in culture medium for the copper loading experiments^30^. We therefore performed preliminary experiments, in which the addition of 100 to 400 micromolar CuCl_2_ in buffered solution or in culture medium to the immortalized keratinocyte cell lines (MK and HK) did not cause any cell death at least for 1 hr incubation under 5% by trypan blue dye exclusion test. For the copper load test, we use 100 μM-CuCl_2_ for 30 min, which is the shortest exposure time to be distinguishable with cytosolic copper retention among several previous experiments^10^,^11^,^14^,^30^. Because we need the good viability of cells on the cell culture inserts after copper exposure as the sample for SR-XRF analysis.

#### Atomic absorption spectroscopy

Organs were dry weighed, nitric acid digested, and analyzed by a Shimadzu atomic absorption spectroscopy AA-6800 (Kyoto, Japan) for Cu, Fe and Zn according to standard protocols. All data was calculated from three individual experiments (two-tailed paired t Tests). The kidney (Kd) and spinal cord (SN) derived from the MD patients and controls were subjected to the measurement of concentration of copper (Cu), iron (Fe), and zinc (Zn) by atomic absorption spectroscopy (AAS, Shimadzu AA-6800). In this experiment, the spinal cord (SN) contains the root nerves, the vessels, and pia mater.

### Statistical analysis

Paired T test is performed by Prism version 5.00 d software (Graphpad software, USA) to compare the elemental concentration between two groups and two regions. Three unaffected individuals are used as controls. The elemental concentrations (mg/kg) are measured by AAS for three times. To compare photon counts, six regions inside each tissue are calculated. Exceptionally, whole posterior root is calculated. All data are analyzed from three individual SR-XRF experiments.

## Additional Information

**How to cite this article**: Kinebuchi, M. *et al*. Diagnostic copper imaging of Menkes disease by synchrotron radiation-generated X-ray fluorescence analysis. *Sci. Rep.* **6**, 33247; doi: 10.1038/srep33247 (2016).

## Figures and Tables

**Table 1:** Cu, Fe, Zn concentrations (photon counts/0.5 sec) in the gut.

	Cu	Zn	Fe
Intestinal Epithelium			
MD (Fig. 5a arrow 1)	13.21 ± 3.85	15.50 ± 4.28	8.86 ± 3.35
Control (Fig. 5c arrow 1)	4.76 ± 1.99	11.63 ± 3.29	15.63 ± 3.71
Intestinal Muscle layer			
MD (Fig. 5b arrow 2)	11.15 ± 3.87	16.98 ± 4.50	18.37 ± 8.36
Control (Fig. 5c arrow 2)	2.99 ± 1.82	8.73 ± 3.39	8.33 ± 2.44
Epithelium of lymph vessel			
MD (Fig. 5b arrow 1)	11.05 ± 4.15	12.18 ± 4.19	10.49 ± 4.27
Control	N.D.	N.D.	N.D.
Vessels in duodenum			
MD vessel 1 (Fig. 5a arrow 2)	26.47 ± 6.68	50.95 ± 8.72	19.42 ± 5.78
MD vessel 2 (Fig. 5a arrow 3)	26.12 ± 6.49	45.47 ± 8.62	19.33 ± 6.19
MD vessel 3 (Fig. 5b arrow 7)	15.87 ± 6.67	27.28 ± 15.65	13.55 ± 4.68
Control vessel	3.23 ± 1.48	8.54 ± 2.96	22.72 ± 11.23
Macrophages in lymph vessel			
MD Macrophage 1 (Fig. 5b arrow 3)	10.59 ± 4.83	10.66 ± 3.90	38.63 ± 11.35
MD Macrophage 2 (Fig. 5b arrow 4)	7.27 ± 3.31	11.26 ± 3.55	11.74 ± 4.74
MD Macrophage 3 (Fig. 5b arrow 5)	9.26 ± 4.42	12.86 ± 4.06	11.73 ± 4.40
MD Macrophage 4 (Fig. 5b arrow 6)	7.26 ± 2.68	9.71 ± 3.07	29.63 ± 7.39
Control macrophage	N.D.	N.D.	N.D.

Mean photon counts (photons/0.5 sec) ± SD (n = 6).

N. D. Not detected.

Control vessel is not shown in Fig. 5c.

I_0_ = 70791.00 ± 457.83.

The condition of SR-XRF analysis and the locations are referred to in Fig. 5.

**Table 2:** Cu, Fe, Zn concentrations (photon counts/0.5 sec) in the kidney.

	Cu	Zn	Fe
Proximal tubular cell			
MD	56.71 ± 11.72	25.42 ± 4.89	21.08 ± 5.26
Control	9.19 ± 1.35	8.53 ± 3.75	2.87 ± 3.34
Distal tubular cell			
MD	9.75 ± 3.18	18.88 ± 4.39	18.53 ± 6.01
Control	9.85 ± 1.31	14.55 ± 5.79	4.54 ± 2.05
Other regions inside cortex of MD			
Bowman’s space	10.75 ± 2.12	11.75 ± 1.67	7.43 ± 2.62
Bowman’s capsule	15.50 ± 4.51	17.89 ± 4.37	61.20 ± 7.30
Mesangium	17.86 ± 10.77	20.26 ± 6.31	55.78 ± 31.56
Gromerulus capillary	15.77 ± 4.11	55.54 ± 7.44	30.42 ± 7.29
Wall of Interlobular artery	17.31 ± 4.15	18.16 ± 4.50	36.30 ± 28.21
Interlobular artery	4.22 ± 2.13	10.07 ± 2.96	4.17 ± 1.97
Other regions inside cortex of control			
Vessel (Fig. 7b arrow 8)	14.49 ± 1.59	58.77 ± 19.67	7.66 ± 5.09

Mean photon counts (photons/0.5 sec) ± SD (n = 6).

N. D. Not detected.

I_0_ = 48604.1 ± 247.574.

The condition of SR-XRF analysis and the locations are referred to in Fig. 7.

**Table 3:** Cu, Fe, Zn concentrations (photon counts/0.5 sec) in the spinal cord, root nerve and veins

	Cu	Zn	Fe
White matter			
MD	3.32 ± 0.28	19.51 ± 4.26	16.24 ± 3.59
Control	5.32 ± 0.32	19.58 ± 5.49	5.45 ± 2.65
Gray matter			
MD	3.48 ± 0.24	14.46 ± 3.41	12.14 ± 2.90
Control	5.34 ± 0.40	15.92 ± 3.93	7.73 ± 2.96
Artery in gray matter			
MD (arrow 3)	3.5 ± 1.98	23.91 ± 4.66	62.91 ± 7.61
Control	6.08 ± 2.81	19.67 ± 3.67	29.63 ± 1.13
Posterior root			
MD	8.95 ± 0.05	30.47 ± 0.19	71.26 ± 0.99
Control	5.65 ± 0.42	8.20 ± 4.97	34.56 ± 3.20
Regions inside posterior root of MD			
arrow 1	17.25 ± 2.99	44.14 ± 1.27	14.6 ± 5.18
arrow 2	16.50 ± 5.45	114.75 ± 5.85	40.56 ± 1.90
arrow 6	54.02 ± 2.68	34.33 ± 1.78	36.00 ± 2.23
arrow 7	12.88 ± 5.11	74.33 ± 8.08	234.6 ± 21.26
arrow 8	51.67 ± 5.14	94.00 ± 6.04	37.63 ± 2.69
arrow 9	112.00 ± 3.68	97.37 ± 3.25	607.00 ± 29.91
arrow 10	17.00 ± 1.99	64.25 ± 2.82	11.34 ± 5.29
Other veins of MD			
Posterior spinal vein (arrow 5)	8.13 ± 2.59	43.40 ± 6.42	104.30 ± 1.72
Vein in dura mater	10.88 ± 2.36	26.75 ± 5.68	76.17 ± 1.93

Mean photon counts (photons/0.5 sec) ± SD (n = 6).

N. D. Not detected.

Two of gaps pointed by arrow 10. Twelve gaps are analyzed.

I_0_ = 99767.6 ± 868.638.

The condition of SR-XRF analysis and the locations are referred to in Figs 8 and 9.
